# Merging adoption of natural climate solutions in agriculture with climatic and non-climatic risks within an (intra)gendered framework

**DOI:** 10.1038/s41598-024-60469-w

**Published:** 2024-05-22

**Authors:** Kwabena Antwi

**Affiliations:** Graduate School of Geography, Clark University, Worcester, MA 01610 USA

**Keywords:** Natural climate solutions, Vulnerability, Climatic risks, Non-climatic risks, Gender, Ghana, Climate-change impacts, Climate-change adaptation

## Abstract

The extant research on climate variability shares significant theoretical contributions to vulnerability and risks. However, the literature mostly focuses on technical solutions to climate extremes which undermines efforts to identify and solve the dynamics within gender groups in using agricultural-based natural climate solutions (NCS) to address climatic and non-climatic risks. With this in mind, this study implements both quantitative and qualitative approaches including household surveys, key informant interviews, and focus group discussions to investigate the adoption of NCS within gender groups to address climatic and non-climatic risks in three selected communities (Katanga, Dakio, and Zonno) in the Bolgatanga East District of Upper East Region of Ghana. The Relative Importance Index (RII) was used to rank the key climatic and non-climatic risks confronting smallholder farmers in the district. Male and female smallholder farmers affirmed that there has been variation in the climate compared to their childhood. The results indicated climate change-induced erosion (*RII* = 0.268) as the highest climatic risk among male smallholder farmers. Increased bushfire (*RII* = 0.263) was the highest climatic risk affecting female smallholder farmers. The findings show that the high cost of farm inputs (*RII* = 0.505) is the highest non-climatic risk among the male smallholder farmers whereas inadequate credit facilities (*RII* = 0.295) affected most of the female smallholder farmers. In adapting to the climatic risks, both male and female smallholder farmers with no formal education plant early maturing crop varieties and cover crops on their farmland. Others engage in traditional non-farm activities such as weaving by using renewable materials with reduced ecological footprints to address non-climatic risks. The male and female smallholder farmers with post-secondary education typically resort to temporal migration during the dry season to work on non-farm jobs. Acknowledging the intra-gendered adoption of NCS among marginalized farming households; not only protects against maladaptation but also improves local-level resilience and climate risk management in Ghana.

## Introduction

The Intergovernmental Panel on Climate Change (IPCC) defines climate variability as fluctuation in the average condition of the climate at geographical and temporal scales beyond that of individual weather events^1^. Ghana is among the majority of sub-Saharan African (SSA) countries that are particularly sensitive to the risks of climate variability^2^. A report by the Environmental Protection Agency (EPA) in 2021 indicates that all of Ghana’s agroecological zones have experienced an increase in temperatures throughout time, along with an overall decrease in rainfall and an increase in irregular rainfall patterns^3^. World Bank^4^ projects that Ghana will experience warming between 2010 and 2050, with the Northern, Upper East, and Upper West regions expected to see the highest temperatures. Future projections indicate that about 1.7 °C to 2.04 °C increase in temperature will be observed in the country. Rainfall will decrease by 2.8%, 10.9%, and 18.6% on average by 2020, 2050, and 2080 respectively across all agroecological zones in the country^5^. This will hamper food security and the attainment of Sustainable Development Goals (SDGs) 1 and 2.

Climatic risks that have an impact on agriculture, human livelihoods, and the environment including extreme drought, unpredictable rainfall amount, higher severity of pests and diseases, and dry spell conditions are particularly common in the Savanna and Sudan agroecological zones of Ghana^6–8^. Tetteh et al.^9^ attributed reduced yield in root and tuber crops like cocoyam and plantain to the effects of climate variability and change on agricultural productivity in the country. Similarly, File et al.^10^ highlighted how the threats of climate change are significantly impacting smallholder farming households by reducing yields of major staple crops such as yam, cassava, sorghum, maize, millet, and rice. Over time, Ghanaian smallholder farmers suffer greatly as a result of these changes due to their dependence on rain-fed agriculture and other ecosystem-related livelihoods. The situation is aggravating household poverty and food insecurity in the Upper East region of the country.

Non-climatic risks such as the high cost of agricultural inputs, poor roads, inadequate irrigation systems, and capital resources, further exacerbate the vulnerabilities faced by smallholder farming households in the Savanna and Sudan agroecological zones of Ghana^11^. For instance, Fagariba et al.^12^ observed that the high cost of inputs such as tractor services, fertilizer, insecticides and weedicides, and the lack of farm labor hinder poor smallholder farmers in northern Ghana from mitigating the challenges associated with climate change. According to Challinor et al.^13^, most smallholder farmers resort to using unimproved groundnut seeds from the local market because of the high cost of improved seeds. A study by Fagariba et al.^12^ showed that smallholder farmers, particularly those in remote areas are not getting enough climate information because of a lack of logistics and inadequate extension services. Smallholder farmers in the region are more vulnerable to both climatic and non-climatic risks because of a lack of infrastructure development, low literacy, and high poverty rates^14^ which exacerbates the effects of climate variability on their livelihoods^15^.

The adoption of agricultural-based natural climate solutions (NCS) to achieve greenhouse gas (GHG) mitigation, conservation, and co-benefits for the environment has gained global attention^16–19^. Natural climate solutions in agricultural landscapes encompass strategies like optimizing grazing, preventing grassland conservation, and implementing approaches on cultivated areas such as reduced tillage, intercropping, cover cropping, and enhanced nutrient management^20,21^. Assan et al.^22^ found a gendered pattern in agricultural-based natural climate solutions adopted by households in Lawra District of the Upper West Region of Ghana. Ndamani and Watanabe^23^ revealed that male and female smallholder farmers are more likely to use crop diversification, irrigation, and agroforestry to mitigate the effects of climate change on their farming activities. Ahmed et al.^24^ also found that even though men are more engaged in farm activities, women in semi-arid Ghana have higher participation in all agricultural activities than men. This proportionate growth of crops by gender indicates agricultural gendered tendencies and alternatives for adapting to climate variability and risks^25,26^. Thus, knowing how differences within gender groups influence smallholder farmers' use of agricultural-based natural climate solutions to reduce climatic and non-climatic risks would also be pivotal when designing adaptation strategies for dryland farming systems. This will give a practical planning guide for more gender-inclusive local-level resilience to climatic and non-climatic risks as well as informed protection against maladaptation.

In this study, I explored the adoption of natural climate solutions in agriculture to address climatic and non-climatic risks within gender groups in the Bolgatanga East District of the Upper East Region, Ghana. Specifically, the study sought to answer the following research questions: (i) What is the social perspective of climate variability in the study area? (ii) Which climatic risks affect smallholder farmers? (iii) Which non-climatic factors hinder agricultural land use dynamics in the district? (iv) How do smallholder farmers in the district use agricultural-based natural climate solutions to address climatic and non-climatic risks in the study area?

## Gender and vulnerability in Ghana

Climate projections indicate that the Guinea savanna agroecological zone of Ghana will see a decrease in mean annual rainfall by 3.5%, 0.9%, and 3.1% in each of the years 2040, 2060, and 2080, respectively. In addition, by 2040, 2060, and 2080, a 1% decline in rainfall is projected across the coastal savannah zone^4,27^. Rainfall variability and frequent droughts, as well as inadequate soil fertility, are major challenges for agricultural production in the zone^28,29^. The Upper West, Northern, and Upper East regions are the most deeply agrarian regions in Ghana where much labor is invested in agriculture^30^. Natural extremes and anomalies in weather conditions caused by climate variability are already reducing crop production, a tendency that is expected to continue as temperatures rise^31^. Arndt et al.^32^ found that climate variability reduces national welfare, with the poor being the most vulnerable.

Ayanlade et al.^33^ highlighted how compound dimensions of vulnerability including migrant status, age, educational level, inequalities of gender, and income affect the risk of climate variability. Amfo and Ali^34^ in their study of cocoa farmers in Ghana found that the training on farm management, the age of cocoa farms, and distance to regional capital shape smallholder cocoa farmers' adaptation to climate variability. Also, the decision-making and resource access of women are marginalized, resulting in female smallholder cocoa farmers having a lower probability of diversifying farm income. This is because male smallholder cocoa farmers typically have better access to capital, land, and climate information than their female counterparts^34^. To the detriment of women, variation in climate adds another layer and makes these disparities worse^35^. For instance, compared to men, women are more susceptible to water shortages, floods, droughts, and heavy rain^36^. They spend more working hours gathering firewood and providing food for their families^37^ while the men are responsible for livestock production^38^. Assan et al.^22^ discovered that during the dry season, female-headed households in the Lawra area of Ghana's Upper West Region either sell cattle, or process shea nuts because they are unable to get the money needed to participate in other subsistence activities like beekeeping and soap production. In contrast, their male counterparts move in pursuit of employment or sell livestock. Sociocultural prejudice makes it difficult for women farmers in the Upper East Region to get land. The inability to secure land tenure has an impact on female smallholder farmer’s capacity to adjust to climate variability and their level of food insecurity increases^25^.

Socioeconomic patterns influence how vulnerable smallholder farmers are to climate variability^7,39^ and differences in behavior between men and women shape their decision-making processes^25^. Several studies including^40–46^ have explored climate shocks, livelihood diversification, and gendered perception of climate variability. Despite this, it remains unclear how the adoption of natural climate solutions in agriculture interacts within gender groups to influence vulnerability to both climatic and non-climatic risks. This study draws a link between the adoption of NCS within gender groups to address climatic and non-climatic risks among smallholder farming households in the Upper East Region of Ghana. Findings from this study highlight the critical importance of understanding intragender-differentiated vulnerability and risk exposure to both climatic and non-climatic risks, as well as the coping capacity of marginalized farming households^24^. In addition, it will serve as a gender-inclusive and context-specific planning guide for policymakers to address the risk of climate variability on dryland farming systems^44^.

## Study design and methods

### Description of the study area

**Figure 1:**
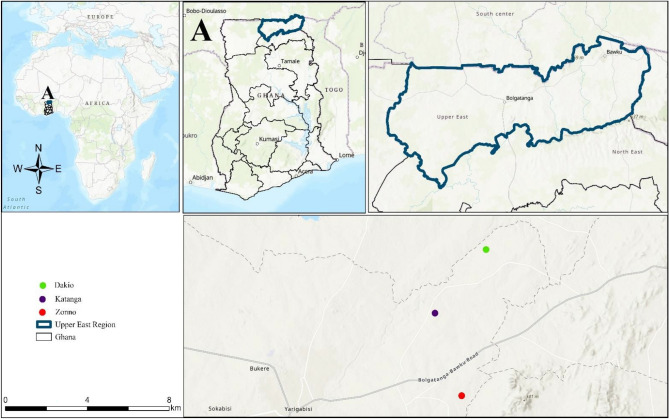
Study area map showing the studied communities (created using ArcGIS Pro 3.2.2; Source: https://www.esri.com/en-us/arcgis/products/arcgis-pro/overview ).

The Upper East Region is one of Ghana’s 16 administrative regions, covering a total land area of 8,842 square kilometers. Situated in the northeastern corner of Ghana, the Upper East region shares its borders with Burkina Faso to the north and Togo to the east (Fig. 1). Geographically, it spans between longitude 0° and 1° 4′′ West and latitudes 10° 15′′ and 10° 10′′ North^47^. The population center of the Upper East Region is situated in its capital, Bolgatanga. The majority of the population, approximately 79%, resides in rural areas, distributed across dispersed settlements^48^. The region experiences an average rainfall of 921 mm, ranging from 645 to 1250 mm. There is a single 5 to 6 months growing season from April/May to September/October, followed by 6 to 7 long dry seasons from October to April. During these periods, characterized by harmattan winds and low humidity, the area is conducive for cultivating horticultural crops such as tomatoes, peppers, onions, and leafy vegetables. From November to mid-February, an extended dry season prevails, marked by cold, dry, and dusty harmattan winds. The natural vegetation consists of savannah woodland, featuring short, resilient trees scattered across the landscape^47^. The soil in the region is mostly formed primarily from granite rocks. It is shallow, lacking in fertility, and has low organic matter, mainly consisting of coarse textures. Despite this, agriculture remains the primary economic activity, employing 80% of the population. The main agricultural produce are millet, guinea-corn, maize, groundnut, beans, sorghum, and dry season tomatoes and onions^47^.

This study focused on three communities in the Bolgatanga East District (Table 1). Established through Legislative Instrument (LI) 2350, the Bolgatanga East District is centered around Zuarungu. Geographically, it shares boundaries with Bongo District to the north, Talensi, and Nabdam Districts to the south and east, and Bolgatanga Municipality to the west in the region. The district spans a total land area of 70.80 square kilometers^30^. The three communities namely Dakio, Katanga, and Zonno were purposively selected for field data collection after consulting some of the agricultural extension officers (AEOs) in the district.

**Table 1:** Characteristics of the studied communities.

	Bolgatanga East District					
	Dakio		Katanga		Zonno	
Sampled households	Males 37 (53)	Females 33 (47)	Males 38 (54)	Females 32 (46)	Males 32 (46)	Females 38 (54)
Main livelihood activity	Crop farming		Crop farming		Crop farming	
Types of farming	Smallholder		Smallholder		Smallholder	
Cultivated crops	Groundnut, soybean, maize, millet		Cowpea, groundnut, sorghum, millet		Millet, maize, groundnut, soybean	

The total sample size is 210, consisting of 107 males and 103 females. The numbers in parentheses indicate the percentage of sampled households in each community.

### Research method

**Figure 2:**
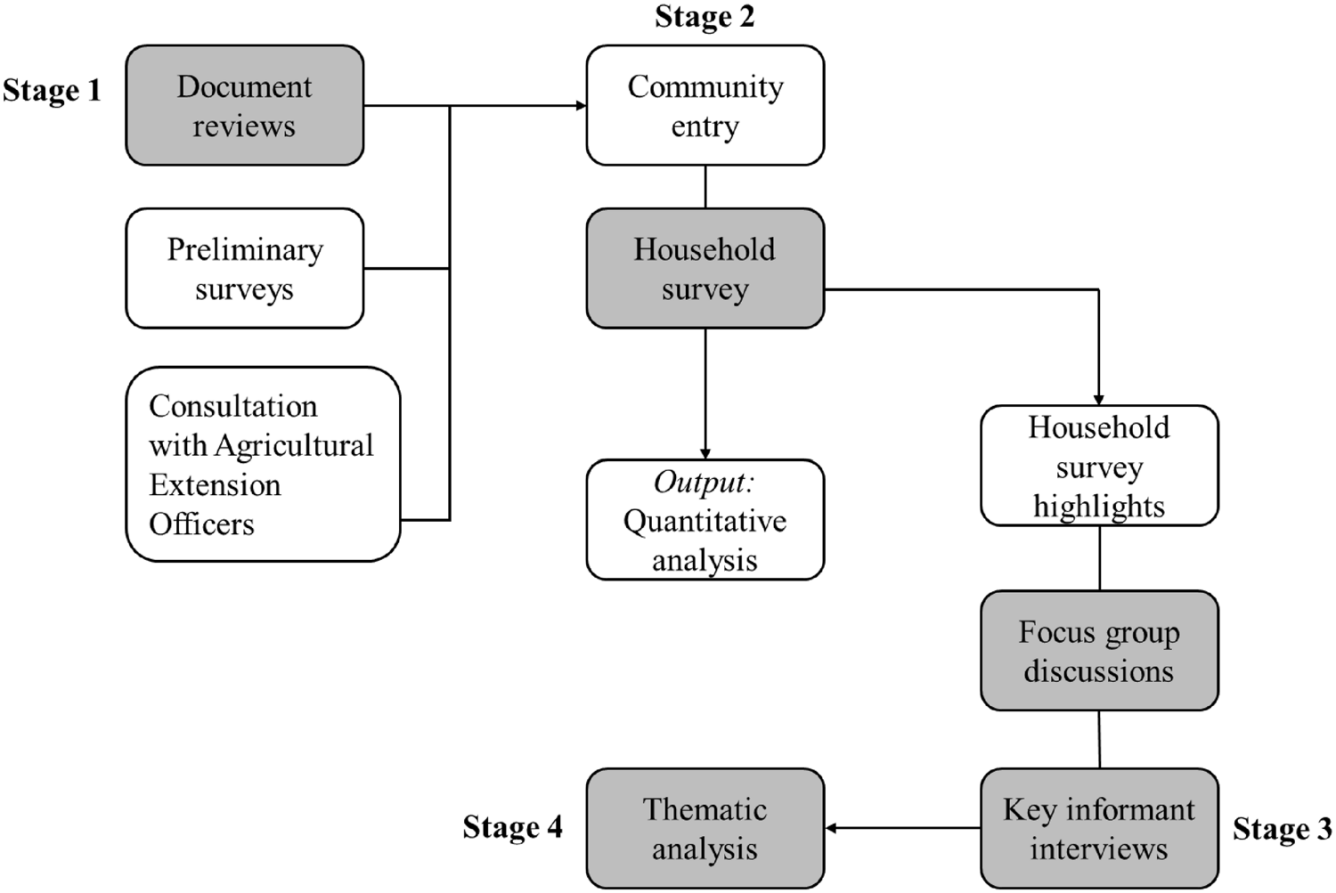
The stages of the research method.

The research involved four (4) stages combining both quantitative and qualitative elements to answer the research questions. Stage 1 entailed document reviews, preliminary surveys, and consultation with agricultural extension officers in the district. Stage 2 involved community entry, household survey, and quantitative analysis (including descriptive analysis and relative importance index). Stage 3 comprised focus group discussions and key informant interviews. Stage 4 involved thematic analysis of the qualitative data from the focus group discussions and key informant interviews (Fig. 2).

The study employed a triangulation research design to increase the validity of the evaluation and research findings. This design was used throughout the research process to address the objectives of the study. Multiple research methodologies, mostly qualitative and quantitative, are used in a single study under the triangulation design to increase the credibility of the findings^49^. The use of the qualitative method helps discover reasons for observed patterns^50^ whilst the quantitative method simplifies the processing and comparison of large amounts of data^51^.

The study analyzed around 64 peer-reviewed publications and institutional reports covering the adoption of natural climate solutions on dryland farming systems, barriers, motivations, and gender mainstreaming in adaptation to climate variability in the region. Using the ethnographic approach, the study focused on analyzing households as the primary unit of analysis^52^. According to Nurani^53^, the approach provides a comprehensive understanding of the phenomena under study from the perspective of the people involved. In each of the study communities, the agriculture extension officers took part in a one-day preliminary survey, and each of them was responsible for leading the community entry. Two hundred and ten smallholder farming households were selected for the study using simple random sampling. The household surveys were conducted in their local language (Gurene) and Asante Twi. Two separate focus group discussions were designed in each study community to enable participants to explore and expand on each other’s responses. This allowed a more common understanding of responses, which improved the reliability of the study findings^54^. Key informant interviews were conducted with a broader group of individuals such as community leaders with appreciable knowledge of agricultural-based natural climate solutions and risks from climate variability to elicit comprehensive responses^55^. With permission granted by each respondent, the interviews were audio recorded.

### Data analysis

Data were coded for analysis using IBM Statistical Package for Social Sciences (SPSS) version 26. A combination of descriptive statistical approaches including frequencies, percentages as well as Relative Importance Index (RII) were used in the study. The Relative Importance Index was used to rank the key climatic and non-climatic risks affecting smallholder farmers in the studied communities^56^. The formula for computing RII is given in Eq. (1).

1 $$\mathrm{RII }=\sum \frac{\left({\text{W}}\right)}{\mathrm{A}} \times {\mathrm{N}}$$

Where: *W* = weight given to a statement provided by a respondent, ranging between 1 and 3 on a 3-scale Likert scale. Here, 1 = very evident, 2 = evident, and 3 = not evident. *A* = highest response integer (3) *N* = total number of respondents considered.

Data collected in this study were initially cleaned by eliminating inaccuracy and replicates using Microsoft Excel Version 2019 to prepare the data for analysis. With the aid of AEAs who are fluent in Gurene, the recorded voices from key informant interviews and FGDs were played repeatedly on mobile phones. Thematic analysis was employed to assess data from the focus group discussions and key informant interviews to identify common themes.

## Results and discussion

### Background characteristics of respondents

The majority of these smallholder farmers were above 50 years old (Table 2). This finding confirms the decline in youth involvement in crop farming in the Upper East Region^30^, possibly attributed to youth migration to southern Ghana for non-farm jobs, aiming to earn money for food, school fees, and health insurance^28^. The majority of the households were dependent on farming although most were males (39.5%) as against (32.9%) females. This finding corroborates reports on the male-dominant land tenure system in the Upper East Region where land inheritance is customarily done through the patrilineal line^57^. This further justifies why male smallholder farmers (39.5%) have more farming experience than female smallholder farmers (27.6%) in the studied communities. However, if the farmland is isolated and deemed to be lacking soil nutrients, female smallholder farmers may have access to it for crop farming^58^. A report by Ghana Statistical Service^30^ indicates that the Upper East Region is one of the most deeply agrarian parts of Ghana where much labor is invested in agriculture. Despite this, unpredictable weather patterns in the region constrain smallholder farmers’ livelihoods.

**Table 2:** Background characteristics of respondents.

Variable	Gender		All
	Male (*n* = 107)	Female (*n* = 103)	(*N* = 210)
State of residency			
Indigene	99 (47.1)	60 (28.6)	159 (75.7)
Not indigene	8 (3.8)	43 (20.5)	51 (24.3)
Household size			
** < **5 individuals	24 (11.4)	27 (12.9)	51 (24.3)
6–10 individuals	50 (23.8)	52 (24.8)	102 (48.6)
> 11 individuals	33 (15.7)	24 (11.4)	57 (27.1)
Age (years)			
< 30	6 (2.9)	12 (5.7)	18 (8.6)
31–40	11 (5.2)	22 (10.5)	33 (15.7)
41–50	22 (10.5)	26 (12.4)	48 (22.9)
> 50	68 (32.4)	43 (20.5)	111 (52.9)
Income source			
Farming	83 (39.5)	69 (32.9)	152 (72.4)
Non-farming	24 (11.5)	34 (16.2)	57 (27.6)
Farming experience (years)			
< 10	7 (3.3)	15 (7.1)	22 (10.5)
10–20	17 (8.1)	30 (14.3)	47 (22.4)
> 20	83 (39.5)	58 (27.6)	141 (67.1)
Marital status			
Single	8 (7.5)	4 (3.9)	12 (5.7)
Married	95 (88.8)	97 (94.2)	192 (91.4)
Divorced	4 (3.7)	2 (1.9)	6 (2.9)
Educational attainment			
No formal	79 (37.6)	64 (30.5)	143 (68.1)
Primary	15 (7.1)	27 (12.9)	42 (20.0)
Secondary	10 (4.8)	9 (4.3)	19 (9.0)
Post-secondary	3 (1.4)	3 (1.4)	6 (2.9)

The numbers in and outside parentheses are percentages and respondent count, respectively.

### Social group perspectives of climate variability in the Bolgatanga East District

When asked if there have been observed changes in the extent of climate variability in the district, male and female smallholder farmers affirmed that there have been variations in the climate compared to their childhood. Responses from the focus group discussions highlighted that climate variability persists in the district. For instance, female farmers in the FGDs at Zonno reported:

“*Nowadays, we cannot predict the rains. It can rain before the farming season begins. Other times, it rains late in the farming season. We prepare the land, and it does not come as expected. This is making it difficult to grow groundnut here*”.

Similarly at Katanga, the participants in the male focus group concurred variation in the climate compared to their childhood. They collectively explained:

"*Temperatures are high. Our small rivers are dried up. It will surprise you to know that the seeds we sow do not germinate because the land is very dry*”.

The findings are supported by those of^59^ and^60^ who highlighted that increasing temperatures with variable rainfall persist in the region. The situation is a major challenge for crop farming because agriculture in Ghana is rainfall-dependent. The variable climate in the district is expected to continue as increasing temperatures and variable rainfall patterns are projected to increase across all agroecological zones in the country^3^. Therefore, using climate information services, early warning systems, and preparedness more effectively is a crucial adaptation strategy to reduce climatic risks from the variable climate in the district^61^.

### Key climatic and non-climatic risks affecting smallholder farmers in the district

Smallholder farmers’ perceptions of rising climatic and non-climatic risks are shared by both male and female smallholder farmers (Tables 3 and 4). From the results, climate change-induced erosion (RII = 0.268) ranked the highest climatic risk among male smallholder farmers in the study communities. This is because the male smallholder farmers clear vegetation and trees from their farms for charcoal production which makes the soil prone to land degradation and erosion. This finding supports studies by Aniah et al.^62^ who associated severe land degradation and soil erosion in the Upper East Region to woodland clearing by farmers. According to Tesfahuneg et al.^63^, the northern Savanna Region of Ghana is the most severely eroded area where lands are damaged by water erosion leading to low soil fertility and destruction of soil structure.

**Table 3:** Gendered vulnerability to climatic risks in the district.

Climatic risks	Very evident (W = 1)		Evident (W = 2)		Not evident (W = 3)		RII			
	Male	Female	Male	Female	Male	Female	Male	Rank	Female	Rank
Climate change-induced erosion	48 (44.86)	38 (36.89)	53 (49.53)	59 (57.28)	5 (4.67)	2 (1.94)	0.268	1	0.257	4
Flooding	53 (49.53)	47 (45.63)	45 (42.06)	43 (41.75)	8 (7.48)	10 (9.71)	0.265	2	0.259	2
Drought	66 (61.68)	74 (71.84)	37 (34.58)	26 (25.24)	4 (3.74)	2 (1.94)	0.241	5	0.210	7
Increased pest infestation	58 (54.21)	63 (61.17)	45 (42.06)	33 (32.04)	4 (3.74)	4 (3.88)	0.254	4	0.224	6
Increased soil nutrient depletion	74 (69.16)	62 (60.19)	28 (26.17)	35 (33.98)	5 (4.67)	6 (5.83)	0.230	7	0.238	5
Increased bushfire	54 (50.47)	50 (48.54)	44 (41.12)	40 (38.83)	8 (7.48)	12 (11.65)	0.263	3	0.263	1
Change in vegetation	60 (56.07)	42 (40.78)	42 (39.25)	53 (51.46)	2 (1.86)	5 (4.85)	0.238	6	0.259	2

W is the weight given to an individual statement provided by the respondents. The numbers in and outside parentheses are percentages and respondent counts. Relative Importance Index (RII) shows rankings of climatic risks.

**Table 4:** Gendered vulnerability to non-climatic risks in the district.

Non-climatic risks	Male	Female	RII			
			Male	Rank	Female	Rank
High cost of farm inputs	106 (99.07)	96 (93.20)	0.505	1	0.467	2
Bad roads to farm	75 (70.09)	77 (74.76)	0.357	6	0.367	6
High fuel cost	94 (87.85)	88 (85.44)	0.448	3	0.419	4
Inadequate agricultural equipment	102 (95.33)	97 (94.17)	0.486	4	0.462	3
Inadequate irrigation facilities	88 (82.24)	85 (82.52)	0.419	5	0.405	5
Labor shortage	69 (64.49)	65 (63.11)	0.329	9	0.310	9
Inadequate ready markets	70 (65.42)	66 (64.08)	0.333	8	0.314	8
Poor access to agricultural extension service	74 (69.16)	68 (66.02)	0.352	7	0.324	7
Inadequate credit facilities	103 (96.26)	99 (96.12)	0.490	2	0.471	1

Relative Importance Index (RII) shows rankings of non-climatic risks. The numbers in and outside parentheses are percentages and respondent counts.

A male smallholder farmer in the focus group discussion at Dakio reported:

“*It could rain up to 3 days in the rainy season. Our farmlands become muddy, and the soil gets soaked in the flood. The flood carries the topsoil away. Our groundnut and Bambara beans are destroyed in erosion*”.

The female smallholder farmers however indicated increased bushfires (RII = 0.263) as the highest climatic risk confronting them. Female smallholder farmers in the district gather shea nuts and produce charcoal by using fire. They are therefore exposed to fire outbreaks more often than male smallholder farmers. The findings of Amoako and Gambiza^64^ showed that women in Ghana’s Northern Regions use fire to minimize grass and lessen snake bites in the woods when gathering shea nuts. Comparatively, the male smallholder farmers reported an increased risk of drought (RII = 0.241) more than their female counterparts (RII = 0.210).

For example, a male smallholder farmer during a key informant interview at Zonno shared his experience in the following report:

“*After sowing, the seeds do not germinate. Some of them rot. Because of the drought, the leaves of the groundnut become dried and difficult to uproot. The drought is affecting our land*”.

Regarding the non-climatic risk, the high cost of farm inputs (RII = 0.505) ranked the highest among male smallholder farmers in the study communities. This finding shows that despite the variable rainfall patterns and high temperatures affecting crop yield in the district, the high cost of farm inputs such as fertilizers and agrochemicals discourage male smallholder farmers in the study communities from cultivating staple crops.

A male farmer narrated the following during the key informant interview at Katanga.

“*We inherited the same farmlands our forefathers used so many years ago. The nutrients in the soil have reduced so we always have to apply fertilizer. If not for the fertilizer, we would not get anything from the farm. But the problem is that the fertilizer is so expensive, and we cannot buy it*”.

The female smallholder farmers however ranked inadequate credit facilities (RII = 0.295) as the key non-climatic risk affecting them. Most financial institutions in the district often charge high-interest rates on credit. Other credit facilities require collateral which most female smallholder farmers are unable to provide because they do not have ownership of physical assets such as land and livestock. This finding corroborates those of Nuhu and Matsui^58^ who highlighted that household adaptation techniques are hindered by financial and crop insurance constraints in Ghana’s Upper East Region.

In a key informant interview at Dakio, a female smallholder farmer narrated:

“*Because of the climate variability, the soil is not giving us more yield as it used to be. Our husbands tell us to apply fertilizer, but we do not have the money to buy and there are no loans for us*”.

Generally, labor shortages and land tenure issues are the least of the non-climatic risks confronting male and female smallholder farmers in the study communities. As Adzawla and Alhassan^65^ indicated, family labor is the basic labor for most subsistence farmers. Therefore, farmers in the district work on the farms of their neighbors. Also, land inheritance in the district is customarily done through a patrilineal system where most lands are owned by male smallholder farmers^58^. Women in the district assume ownership of farmland upon the death of a husband.

### Adoption of agricultural-based natural climate solutions

**Figure 3:**
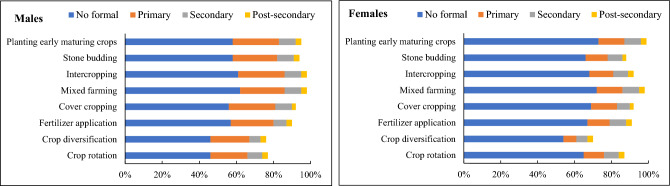
Educational level and the adoption of natural climate solutions among male and female smallholder farmers.

**Figure 4:**
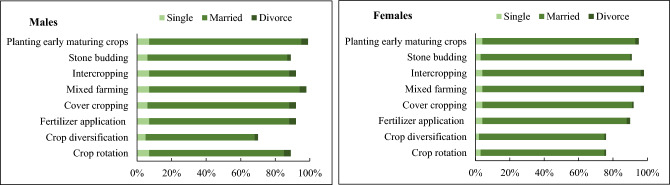
Marital status and the adoption of natural climate solutions among male and female smallholder farmers.

**Figure 5:**
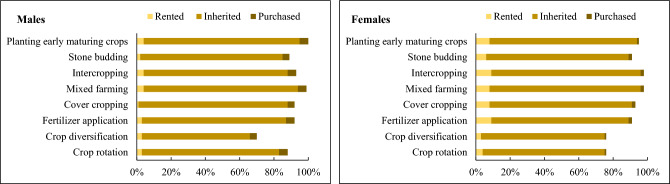
Land tenure system and the adoption of natural climate solutions among male and female smallholder farmers.

The choice of using agricultural-based natural climate solutions to address the risks of climate variability within gender groups is highlighted in Figs. 3, 4, and 5. Generally, the results indicated that male and female smallholder farmers with no formal education use agricultural-based natural climate solutions such as crop rotation, crop diversification, and stone budding more than those with secondary and post-secondary education. The females with no formal education particularly plant early maturing crops than the male farmers on their rental lands because of the patrilineal system in the region which inhibits land ownership for women. Fosu-Mensah et al.^66^ reported similar findings in the Sekyedumase district of the Ashanti region, Ghana, indicating that educational level does not have a significant impact on smallholder farmers' adaptation to climate variability. A study by Asare-Nuamah and Amungwa^67^, however, revealed contrary results stating that an increase in education increases smallholder farmers' adaptation to climate change.

In addition to educational levels, marital status influenced the choice of smallholder farmers to use natural climate solutions. In particular, married female farmers use intercropping and early maturing crop varieties on their farms more than single and divorced female farmers. This finding supports those of^37^ who asserted that married female farmers use climate-smart agricultural (CSA) interventions on their farms more than single and divorced female farmers. Sarfo et al.^68^ for instance, concluded that in a traditional Ghanaian community, married men are responsible for protecting and providing for their families. Thus, they use agricultural-based climate solutions to reduce the risks of climate variability than single and divorced male farmers. According to Ayamga et al.^69^, the farmland tenure system influences smallholder farmers’ on-farm investment decisions for adaptation. The findings in this study substantiate^69^. Both male and female farmers with inherited farmland tenure systems use crop diversification, crop rotation, and intercropping more on their farms than those with rented and purchased farmlands. This latter observation corroborates the work of Ghebru and Lambrecht^70^, who drew attention to the fact that farmlands acquired through inheritance are more highly secured than those acquired through gifts from family and friends. Therefore, male and female smallholder farmers with inherited farmlands diversify their crops to generate family income. By using crop diversification, crop rotation, and intercropping on the farm, male and female farmers with inherited farmlands can reduce the risks associated with relying on a single crop. If one crop fails due to climatic risks from climate variability including pests and diseases, the other crops may thrive, ensuring some level of harvest and income for the household. Crop rotation is of particular interest in most Ghanaian farming communities because it helps to maintain soil fertility for crop productivity.

Non-farm livelihood diversifications including pito brewing, charcoal production, and small-scale mining serve as an essential non-climatic risk-spreading strategy in smallholder farming households^71^. Although most of these non-farm livelihood activities guarantee short-term income for farming households, they generally harm the environment. For instance, charcoal production and small-scale mining result in deforestation, land degradation, and water pollution^72^. In addressing this, female smallholder farmers with no formal education engage in traditional non-farm activities such as weaving, and petty trading. These traditional activities often involve renewable materials such as cotton and minimal chemical inputs which reduce the ecological footprints associated with production. The male smallholder farmers resort to using scrap metals in blacksmithing and depending on family and friends. Those with post-secondary education rely on temporal migration during the dry season to spread the risk of crop failure. These traditional activities align with principles of environmental stewardship and generate a complementary source of earnings to meet the basic needs of the households. Efforts have been made by non-governmental organizations (NGOs) such as World Vision International, Care International, and Action Aid to improve smallholder farmers’ adaptation to non-climatic risks in the district. These NGOs represent the voice of marginalized smallholder farmers to increase their food and nutrition security as well as resilience to non-climatic emergencies. For instance, World Vision International provides loans for shea nut extraction and petty trading. Others such as ProNet and Care International provide farm inputs and entrepreneurial training programs for women^73^.

## Conclusion and policy implications

The study confirmed increasing awareness of climate variability among male and female smallholder farmers in the Bolgatanga East District of the Upper East Region of Ghana. Male and female smallholder farmers emphasized their vulnerability to climatic and non-climatic risks. The study revealed that climate change-induced erosion is the highest climatic risk affecting male smallholder farmers. Increased bushfire was the highest climatic risk affecting female smallholder farmers. Also, the high cost of farm inputs ranked the highest non-climatic risk among the male smallholder farmers whereas inadequate credit facilities affected female smallholder farmers. In addressing the climatic risks, male and female smallholder farmers with no formal education use natural climate solutions such as crop rotation, and crop diversification more than those with secondary and post-secondary education. Further comparison showed that married male and female farmers use intercropping and early maturing crop varieties on their farms more than single and divorced female farmers to minimize the risks of climate variability. The design of climate risk management must consider exploring within-gender-specific needs that address constraints affecting smallholder farmers' vulnerability to climate variability. By mainstreaming intra-gendered adoption of agricultural-based natural climate solutions among male and female smallholder farmers to address climate variability, the local-level resilience and climate risk management in Ghana could be improved.

## Limitations and suggestions for future research

A key limitation of this study is the sparse distribution of houses in the region, making data collection challenging. The district’s population is notably smaller considering its geographical expanse, largely due to families migrating to southern Ghana for non-farm activities. In addition to highlighting key findings, future research can explore strategies for scaling up gender-inclusive adoption of natural climate solutions in agriculture. This might involve examining successful case studies or pilot projects that have effectively promoted the adoption of natural climate solutions among both male and female smallholder farmers. Additionally, examining the availability and effectiveness of gender-sensitive extension services and training programs for the adoption of natural climate solutions in agriculture could be a crucial avenue for further exploration.

## Data Availability

The datasets used and/or analyzed during the current study are available from the corresponding author on reasonable request.
